# The Response of Synthetic Adrenocorticotropic Hormone (ACTH) Treatment in Pediatric Drug-Resistant Epilepsy Other Than Infantile Epileptic Spasms Syndrome: A Retrospective Observational Study

**DOI:** 10.7759/cureus.46431

**Published:** 2023-10-03

**Authors:** Hajar Alammar, Ahmed Al-Rumayyan, Duaa Baarmah, Muhammad T Alrifai

**Affiliations:** 1 Neurology, King Saud Bin Abdulaziz University for Health Sciences College of Medicine, Riyadh, SAU; 2 Pediatric Neurology, King Saud Bin Abdulaziz University for Health Sciences College of Medicine, Riyadh, SAU; 3 Pediatric Neurology, King Abdullah Specialized Children Hospital, Ministry of National Guard Health Affairs, Riyadh, SAU

**Keywords:** drug-resistant, epilepsy, long-term outcome, short-term outcome, adrenocorticotropic hormone (acth)

## Abstract

Introduction: Adrenocorticotropic hormone (ACTH) is a tropic hormone naturally secreted by the anterior pituitary gland to stimulate the secretion of cortisol and androgens. ACTH is used in non-tuberous sclerosis infantile epileptic spasms syndrome (IESS), and it has shown significant, promising results in epilepsy syndromes with possible inflammatory processes. However, many studies have also demonstrated a promising potential even in other types of drug-resistant epilepsy.

Material and method: This study is a retrospective observational study that follows the clinical characteristics and outcomes of nine pediatric patients with drug-resistant epilepsy treated with short-term synthetic ACTH in Saudi Arabia. The response was assessed during the ACTH infusion and after three months.

Results: During infusion, six of the nine (66%) patients had a short-term (within two weeks) favorable response, with a more than 50% reduction in seizure frequency. Four of the nine (44%) patients had complete responses with seizure freedom. After three months, four patients (44%) had a three-month seizure frequency reduction of more than 30% attributed to ACTH, including one patient with an IESS history who had a 70% reduction in seizure frequency. Of the four patients who had a complete response, three (75%) had a seizure relapse after tapering in the following three months.

Conclusion: This case series adds to the literature to suggest ACTH treatment of drug-resistant epilepsies other than IESS might benefit some patients in the acute setting but they are less likely to maintain a sustained treatment response. Randomized and large sample size studies are necessary to assess treatment response and accurately aid in appropriate patient selection.

## Introduction

Epilepsy is a group of neurological disorders that predispose the brain to recurrent epileptic seizures [1]. Practically, it is defined as at least two unprovoked seizures occurring more than 24 hours apart, one unprovoked seizure with a risk of another of more than 60%, or diagnosis of an epilepsy syndrome, and it is considered to be resolved if an individual is seizure-free for more than 10 years with five years off anti-seizure medications, or if it is an age-dependent syndrome and they pass the applicable age [2]. It is considered one of the most common neurological conditions, affecting 70 million individuals worldwide, including 3.2-5.5/1,000 children in developed countries and 3.6-44/1,000 in underdeveloped countries [3,4]. Despite the availability of various anti-seizure medications, it is estimated that 20-30% of patients do not respond to treatment [5]. Drug-resistant epilepsy is the failure to control seizures after a trial of two well-tolerated, appropriately chosen, and adequately tried anti-seizure medications (ASMs), whether as monotherapy or combined [6,7]. Multiple alternative treatments have been used, including surgery, neuromodulation, ketogenic diet, immunoglobulins, and hormonal therapy, including oral steroids and adrenocorticotropic hormone (ACTH). Few studies have addressed the use of corticosteroids in drug-resistant epilepsy. A recent systematic review found only 15 studies with a combined 436 patients with pooled analysis of a seizure reduction in 50% in both pediatric and adult patients, besides seizure freedom in 15% of the patients [8]. Nevertheless, the use of ACTH in drug-resistant epilepsy aside from infantile epileptic spasms syndrome (IESS) is still not clearly established [9].

ACTH is a tropic hormone that is naturally secreted by the anterior pituitary gland to stimulate the secretion of cortisol and androgens [10]. Furthermore, the hypothalamic-pituitary axis controls its secretion, which is responsible for the neurophysiologic response to stressors [11]. ACTH, besides steroids and vigabatrin, is a first-line treatment for non-tuberous sclerosis IESS, which is classically resistant to other anti-seizure medications [12]. Many mechanisms have been theorized for the antiepileptic effect of ACTH. However, the exact mechanisms of action remain uncertain [11]. The suggested mechanisms include the stimulation of glucocorticoids that improve blood-brain barrier integrity and alter brain cells’ electrical activity by increasing the influx of calcium [13,14]. Another mechanism of ACTH is through the downregulation of corticotropin-releasing hormone (CRH) from the hypothalamus, which has a proconvulsant effect [15]. In addition, it enhances the synthesis of precursors of neuroactive steroids that modulate g-aminobutyric acids (GABA) receptors such as dihydroxy-corticosterone (DHDOC) and tetrahydrodeoxy-corticosterone (THDOC), thereby affecting the cells’ excitability [16].

ACTH has shown significant, promising results in epilepsy syndromes with possible inflammatory processes such as Landau-Kleffner and Lennox-Gastaut syndromes and Rasmussen’s encephalitis [13]. However, many studies have demonstrated a promising potential even in other types of drug-resistant epilepsy. Epilepsy with myoclonic atonic seizures (EMAtS), formerly known as Doose syndrome, is commonly treated with a ketogenic diet; however, hormonal therapy, including ACTH, showed improvement in resistant cases [17]. It is essential to study the effectiveness of ACTH treatment to help establish a guideline when treating intractable seizures. Therefore, this study seeks to report the experience of a single pediatric epilepsy center utilizing ACTH for drug-resistant pediatric epilepsy in King Abdulaziz Medical City in Riyadh, Saudi Arabia.

## Materials and methods

Data were retrospectively collected from electronic medical records by the author and medical student data collectors from patients admitted to King Abdullah Specialist Children’s Hospital for drug-resistant epilepsy. They received ACTH treatment from January 2015 to December 2020. Nine patients were identified. The data collected include demographic data, developmental status, seizure type, epilepsy syndrome, radiological features, electroencephalogram (EEG) findings, etiology, anti-seizure medications used, ACTH therapy, and response to treatment. Synthetic ACTH (tetracosactide) was used for treatment via intravenous (IV) route during admission and intramuscular (IM) route as an outpatient for weaning of therapy. IV ACTH was administered as an inpatient treatment. The dosing equivalent of synthetic ACTH to natural ACTH is 0.01mg = 1 IU. The dose was chosen to give an equivalent dose to natural ACTH at 150 IU/m2 for two weeks, followed by a weaning dose given as an outpatient via the intramuscular route as follows: 30 IU/m2 daily for two days, 15 IU/m2 daily for two days, 10 IU/m2 daily for two days, and 10 IU/m2 every other day for two doses, then discontinued. The response was assessed within two weeks of IV treatment. Sometimes a shorter course of treatment was used if no response was observed according to the discretion of the treating physician and the request of the family. Response to therapy was estimated based on seizure frequency reported by parents and primary nursing staff, documented in medical records during infusion and after three months. No Identifying information was collected, and patients’ confidentiality was preserved. IRB approval (reference number IRBC/1574/19) was obtained from King Abdullah International Medical Research Center (KAIMRC) prior to any records retrieval.

## Results

The study includes nine patients with drug-resistant epilepsy, two females and seven males. The mean age was 6.6 years. Most patients had an intellectual disability or developmental delay based on neuropsychological clinic assessment medical records (three severe, one moderate, and three mild cases). Seizure semiology and etiology were diverse among patients. Semiology types were various, and most patients had more than one semiology, including generalized tonic-clonic seizure, focal impaired awareness seizure, focal aware seizure, atonic seizure, attacks of apnea and unresponsiveness, status epilepticus, and in two patients, electrical status epilepticus in sleep (ESES), defined by the clinical finding of deterioration of development/language with a spike wave index of more 85%. Etiologies included hypoxic-ischemic injury (two of nine), genetic (three of nine), post-trauma (one of nine), and unknown causes (three of nine). One patient had a history of IESS but none of the patients had an epilepsy syndrome diagnosis or a history of West syndrome. All patients had abnormal EEG, and seven had imaging abnormalities (Table 1). Most patients were on three or more ASMs prior to treatment. 

**Table 1:** Patients’ clinical information. ID: intellectual disability; GDD: global developmental delay; GTCS: generalized tonic–clonic seizure; FIAS: focal impaired awareness seizure; FAS: focal aware seizure; ESES: electrographic status epilepticus in sleep; SE: status epilepticus; HS: hemisphere; SENH: subependymal nodular heterotopia; PMG: polymicrogyria; BG: background; EEG: electroencephalogram; GSWD: generalized spike-wave discharge

Patient	Sex	Age	Developmental status	Seizure type	Etiology	MRI	EEG ictal	Interictal EEG	EEG post-therapy
1	female	11 years	mild ID	atonic, tonic, and GTCs	unknown	unremarkable		bifrontal discharges	NA
2	female	5 years	severe GDD	FIAS	hypoxic-ischemic injury	multiple infarcts and global atrophy		slow BG, BL central discharges	slow BG, no seizures
3	male	5 years	mild ID	FIAS, atonic, GTCS	X-linked chondrodysplasia punctata	right frontal PMG; bilateral SENH		slow BG, GSWD	no changes from the previous EEG
4	male	11 years	severe ID	atonic, FIAS	early infantile epileptic encephalopathy type 37	unremarkable	left central focal seizures	slow BG multifocal spike and wave discharges	slow BG, multifocal spike and wave discharges
5	male	11 years	severe ID	left HS electrographic SE and ESES	post-traumatic hydrocephalus	hydrocephalus and severe thinning of the white matter bilaterally	left HS electrographic SE	ESES	no changes from the previous EEG
6	male	7 years	mild developmental regression (at age 3)	GTC, atonic, absence, FAS	unknown	unremarkable		bifront-central discharge	no changes from the previous EEG
7	male	7 years	moderate ID	drop attacks, FIAS, GTCS, ESES	unknown	hypoplastic cerebellum		ESES	normal EEG
8	male	32 months	none	FIAS	hypoxic-ischemic injury	watershed infarct	right frontal seizures	right frontal and temporal discharges	normal EEG
9	male	7 months	moderate GDD	attacks of apnea and unresponsiveness	QARS gene homozygous variant	delayed myelination		multifocal discharges	Slow BG, multifocal epileptiform discharges, ES

The average duration of treatment was 19.7 days, with a mode of 22 days (Table 2). No adverse events of treatment were documented in the medical records of any of the patients, and for none of them was the medication stopped due to serious effects. Responses to ACTH were assessed by seizure frequency during ACTH infusion and during the three months following administration. During infusion, six of the nine patients (66%) had a favorable response during infusion, with more than a 50% reduction in seizure frequency. Four of the nine patients (44%) had complete responses with seizure freedom. After three months, of the four patients who had a complete response, three (75%) had seizure relapse after tapering. Two had a relapse of previous types of seizure, and one developed a new type of seizure (absence). Three patients out of the nine continued to have significant improvement (more than 30%) in seizure frequency attributed to ACTH administration (Table 2). While Patient 8 became seizure-free after three months, it was not attributed to ACTH but rather to lacosamide, since he showed no response during infusion but responded after adding lacosamide. On the other hand, Patient 9 was also started on phenobarbital and phenytoin, but improvement could be attributed to ACTH since improvements were noticed during ACTH infusion.

**Table 2:** ACTH treatment course. * Induction dose divided every 12 hours; **total duration of full dose and weaning doses ACTH: adrenocorticotropic hormone; ASM: anti-seizure medication; EEG: electroencephalogram; ESES: electrical status epilepticus in sleep

Patient	ASMs prior to ACTH treatment	ACTH dose (mg)*	ACTH dose (IU/m^2^)	Treatment duration (days) **	Frequency of seizure before therapy	Percent of reduction in seizure during infusion	Seizure relapse after ACTH therapy	Percent reduction in seizure frequency in the following three months
1	phenobarbital, lamotrigine, and rufinamide	1	150	22	5–7 times/day	60%	none	30%
2	topiramate, carbamazepine, valproic acid, phenobarbital, vigabatrin, and levetiracetam	0.39	150	22	20 times/day	85%	none	70%
3	valproic acid, rufinamide, and levetiracetam	0.5	150	22	4–5 times/day	100%	present	<20%
4	levetiracetam, ethosuximide, phenobarbital, and clobazam	0.5	150	22	>20 times/day	100%	present	seizures recurrence after three months of new type: absence
5	valproic acid and levetiracetam	0.5	150	29	ESES	<20%, no improvement in EEG	-	<20%
6	carbamazepine, ethosuximide, and levetiracetam	0.25	105	7	15–20 times/day	<20%	-	<20%
7	topiramate, levetiracetam, and clonazepam	0.63	150	22	15 times/day and ESES	100%	none	60%
8	valproic acid, carbamazepine, levetiracetam, and topiramate	0.25	91	10	4–6 times/day	<20%	-	100% could be attributed to lacosamide
9	phenobarbital, levetiracetam, and phenytoin	0.2	150	22	8–10 times/day	100%	present	100% could be attributed to multiple medications

## Discussion

During the initial phase, the majority of patients (six of nine; 67%), had seizure reduction greater than 50% and four became seizure-free (44%). At three months, the number of patients with sustained greater than 50% seizure reduction was three of nine (33%) with most patients having a recurrence of one or more types of seizures These data suggest that ACTH might be effective in the acute phase but is unlikely to be a sustained response in most cases. The results of this study show a considerably similar pattern to other literature. In a recent study by Nasiri et al., of 25 children with severe resistant epilepsy, 72% (18/25) achieved appropriate responses and a more than 50% reduction in seizure frequency after using ACTH treatment. However after three months 38% (seven of 18) had seizure reoccurrence [18]. In a retrospective assessment by Okumura et al. of 15 patients with generalized epilepsy who received ACTH, 13 of them had seizure freedom, and two were partially responsive to the treatment, with recurrence in six patients within three months suggesting a transitory improvement effect of ACTH [19]. These findings could suggest the usage of ACTH for abrupt seizures in the acute setting rather than long-term treatment.

Similarly, multiple case report studies reported improved seizure frequency. A patient with history of atypical absence seizure received short-term ACTH. He achieved seizure freedom in a few days with recurrence after one month of therapy termination. He was started on long-term weekly ACTH for one year with favorable response in seizure frequency, EEG and developmental status [9]. Another case report of a patient with refractory hemiconvulsion-hemiplegia-epilepsy (HHE) syndrome was controlled with ACTH but another type of seizure emerged soon after [20]. A case report of two patients reports significant improvement with one patient becoming seizure-free with no relapse in the following six months [21].

Nevertheless, the lack of controlled studies prevents any reliable conclusion. A systematic review of randomized clinical trials that investigated the use of corticosteroids, including ACTH, for childhood epilepsy other than epileptic spasms found only a single trial [22]. The trial was a crossover, double-blinded randomized controlled trial of three children who received ACTH compared to placebo; the overall reduction was more than 25% with GRADE working group grades of low-quality evidence [22,23]. In our study, one of two patients with electrical status epilepticus during slow-wave sleep (ESES) had improved EEG abnormalities. While treatment of ESES is still challenging, a pooled analysis of retrospective studies found steroids to be more effective than benzodiazepines and ASMs in both cognitive and EEG improvement [24,25]. Regarding ACTH, a prior study that used ACTH among other strategies to treat ESES found that ACTH had a temporary improvement [26].

Limitations

While this small case series shows promising potential, it has considerable limitations that restrict the control of potential biases and hinder drawing any conclusion. These limitations include the small sample size, the heterogeneous nature of the patients’ conditions and treatment regimens, and the study’s observational and retrospective nature. Additionally, the data were limited to electronic medical records documentation. Multiple ASMs were started simultaneously or close to the ACTH therapy, making isolating its effects or attributing response difficult. In addition, hospitalization use of IV medications could contribute to a placebo effect along with other possible confounders. This study and other studies give more insight into this therapy with good potential in this very difficult-to-control epilepsies. In order to clarify the effectiveness of this therapy modality, further studies with larger sample sizes and prospective randomized control methodology are needed. Furthermore, we recommend longer-duration therapy or intermittent monthly pulses for future studies in order to maintain response and decrease the chance of recurrences. 

## Conclusions

This case series supports the consideration of ACTH as a non-conventional treatment of drug-resistant epilepsy other than IESS. However, it might benefit some patients in the acute setting but are less likely to maintain a sustained treatment response. Randomized and large sample size studies with longer duration are necessary to assess treatment response and accurately aid in appropriate patient selection.
